# Effect of Additional Roles Reimbursement Scheme roles on prescription patterns and patient satisfaction in England: a retrospective panel data analysis

**DOI:** 10.3399/BJGP.2024.0196

**Published:** 2024-12-10

**Authors:** Catia Nicodemo, Chris Salisbury, Stavros Petrou

**Affiliations:** Nuffield Department of Primary Care Health Sciences, University of Oxford, Oxford, UK; professor of health economics, Brunel University of London, London, UK.; Centre for Academic Primary Care, University of Bristol, Bristol, UK.; Nuffield Department of Primary Care Health Sciences, University of Oxford, Oxford, UK.

**Keywords:** allied health care, ARRS, general practice, prescriptions, satisfaction, retrospective studies

## Abstract

**Background:**

In 2019, the Additional Roles Reimbursement Scheme (ARRS) was introduced in England as a crucial component of the government’s manifesto pledge to enhance access to general practice. The primary objective was to recruit 26 000 extra personnel through new roles into general practice.

**Aim:**

To analyse the effects of ARRS staff on prescription rates and patient satisfaction.

**Design and setting:**

A retrospective panel data analysis combining data from the General Workforce Minimum Dataset and NHS Digital datasets about primary care practices and their activity from 2018 to 2022. The study included data from >6000 general practices.

**Method:**

A linear regression analysis was conducted to determine the association between ARRS staff and prescription rates and patient satisfaction, controlling for patient and practice characteristics.

**Results:**

The results showed that ARRS roles tend to be more frequent in larger general practices, with fewer full-time GPs per patient, and with more overseas trained GPs. The use of ARRS staff was significantly associated with lower prescription rates (β = −0.52, *P*<0.001) and higher patient satisfaction (β = 3.2, *P*<0.001), after controlling for patient and practice characteristics.

**Conclusion:**

This study suggests that the ARRS has the potential to have a positive role in primary care, notably through reduced prescription rates and improved patient satisfaction. Further research is needed to explore the long-term effects of the ARRS on primary care, including patient outcomes and healthcare costs, and the potential barriers to its implementation.

## Introduction

Increasing workforce pressures in general practice are being addressed, in part, by employing practitioners without medical training, commonly referred to as additional role practitioners. These include qualified non-medical health professionals operating at an enhanced level (for example, paramedics, clinical pharmacists, and first-contact physiotherapists) and professionals undertaking clinical roles less familiar in the UK (for example, physician associates).^1^ Categorising these practitioners is challenging because of overlapping responsibilities. In addition to those directly employed by GP practices, primary care networks (PCNs) have had access to funding through the Network Contract Directed Enhanced Service Additional Roles Reimbursement Scheme (ARRS) since April 2020. This scheme supports the employment of certain additional role practitioners, whose deployment is shared between the PCN’s member GP practices. Reimbursement eligibility covers a wide range of roles (see Supplementary Table S1).^2^

Although in some cases ARRS professionals may alleviate the workload of GPs by undertaking routine tasks, the scheme’s broader objective is also to integrate advanced practitioners with specialised capabilities that complement and enhance the services offered by GPs. Some advanced practitioners possess expertise gained from working in specialist hospital clinics and many provide specific skills such as in emergency care or rehabilitation, offering a different range of expertise from that usually offered by GPs. Moreover, ARRS professionals contribute to chronic disease management, medication reviews, and social prescribing support for patients with non-medical needs. Overall, policymakers view the integration of allied health and social care professionals into PCNs through the ARRS as a crucial step towards achieving more patient-centred and efficient primary care services in England, but the scheme remains controversial.

The use of these new roles in primary care is still in its early stages, and there is limited evidence of their long-term impact. Moreover, the available literature lacks definitive evidence regarding the impact of incorporating new roles into primary care, with much of the current research focusing on specific professional types, or a narrow set of outcomes, and with a focus more on organisational impact.

**Table:** How this fits in

The Additional Roles Reimbursement Scheme (ARRS) has been introduced to increase access to general practice, but its impact on clinical workload, health outcomes, quality indicators, and patient satisfaction is unclear. This cross-sectional study analysed data from >6000 primary care practices and found that ARRS roles tend to be hired in large practices, and practices with a shortage of GPs. The study suggests that ARRS-funded workers have the potential to reduce prescription rates and increase patient satisfaction. This information should inform workforce deployment decisions by primary care commissioners and GP practice managers.

This study aimed to investigate the factors associated with the presence of ARRS roles in general practices, and specifically whether ARRS roles are more likely to be present in practices that are already well staffed or conversely in practices with a shortage of GPs. The study aimed to provide insights into the potential effects of the ARRS on prescription rates and patient satisfaction.

## Method

### Study design

The study design involved a retrospective panel data analysis of England-wide quarterly data at the general practice level covering the period March 2018 to March 2022.

### Data

In this study, data were utilised from various sources to investigate the impact of the ARRS on primary care outcomes. The primary data source was the NHS Digital National Health Applications and Infrastructure Services/‘Exeter’ GP payment system that provides information on the workforce present in each practice (encompassing GPs, practice nurses, ARRS-reimbursed roles, and administrative roles, for example).^3^ The data were collected quarterly by financial year, and the study focused on the period between January 2018 and December 2021. NHS Digital imputes missing data for non-reporting practices or those with incomplete survey responses. To ensure data reliability, only practices with complete information were included in the study.

By using the general practice code, these data were linked with other datasets through the practice code for all practices, including:

- the General Practice Patient Survey;^4^
- the Quality and Outcomes Framework (QOF);^5^
- prescribing data;^6^
- the Index of Multiple Deprivation score at the general practice level, 2019;^7^ and
- the PCN workforce database.^8^

Further information about these databases are provided in Supplementary Table S2. The analysis examined practice-level outcomes related to practices and patients. Specifically, for practice-level outcomes, the key predictor variable was the additional clinical capacity added through the ARRS, measured by full-time equivalent (FTE) ARRS-funded staff. The study captured the number of FTEs of ARRS roles employed directly within each practice during the study period. However, because ARRS hiring can also occur centrally through PCNs with personnel who are then deployed across constituent practices, the current study conducted a secondary analysis where the authors assumed that ARRS staff funded centrally were equally shared between practices in the PCN.

The ARRS roles identified in the data and which defined the study’s main predictor variable included: clinical pharmacists, pharmacy technicians, social prescribing link workers, health and wellbeing coaches, care coordinators, physician associates, first-contact physiotherapists, dietitians, podiatrists, occupational therapists, mental health practitioners, paramedics, and nursing associates. However, the highest FTE staffing levels are provided by clinical pharmacists (by far the most common role), paramedics, and first-contact physiotherapists (as presented in Supplementary Table S3).

Two measures were created to quantify additional staffing capacity generated by the ARRS:

- practice ARRS roles: the total FTE counts of new clinical roles hired directly by each general practice using ARRS reimbursement funding. This represented practice-level decisions to expand on-site staff; and
- practice plus PCN-hired ARRS roles: this encompassed the FTE counts of roles hired by each practice as above, plus a share of any new ARRS roles that the practice’s associated PCN hired centrally. This estimate reflected the total ARRS staffing capacity available across both practice and PCN levels.

### Outcomes

Primary outcomes of interest included prescription rates (number of items prescribed over total patients in practices for all prescriptions, mental health medications, statins, analgesics, and antibiotics) derived from NHS prescription data (NHS 2021), and overall patient satisfaction and satisfaction with management of chronic diseases (number of people who answered that they were ‘satisfied’ or ‘very satisfied’ with their practice and the management of chronic diseases within their practice) derived from the General Practice Patient Survey.^4^^,^^6^

### Analysis

Ordinary least square (OLS) regression was used to estimate associations between the FTE counts of ARRS roles in each practice and the following practice-level factors: the number of FTE GPs per 1000 registered patients, the number of FTE nurses per 1000 registered patients (excluding nursing associates), the proportion of overseas trained GPs (number of overseas trained GPs over the total number of GPs in the practice), the proportion of female GPs (total female GPs over the total GPs in the practice), and the number of patients weighted at practice level (the weighted population at practice level is based on the formula used to allocate health resources and considers age–sex weightings, health inequality adjustments, location cost differences, and higher service costs in remote/sparse areas).^9^ The study controlled for prevalence of 12 diseases that are included in the QOF scheme (asthma, cancer, depression, diabetes, hypertension, chronic kidney disease, chronic obstructive pulmonary disease, cardiovascular disease, dementia, epilepsy, mental health, and heart failure). Finally, the study controlled for clinical commissioning group (CCG), year, and quarterly fixed effects. These factors were chosen to enable the authors to explore the relationship between where ARRS roles were employed and where they are most likely to be needed.^10^^,^^11^

The second set of analyses explored how the FTE counts of ARRS roles in primary care affected prescribing rates and patterns and patient satisfaction. An OLS model was used with prescribing data, for the same study period, to estimate the number of items prescribed per practice per quarter for: total prescriptions, mental health medications, statins, analgesics, and antibiotics. These medications were chosen because it was hypothesised that greater provision of staff providing better access to care and a wider range of support might have a particular impact on these types of medication.

In a separate analysis, patient satisfaction extracted from the General Practice Patient Survey was used to examine whether FTE counts of ARRS roles in a practice had an impact on overall satisfaction and satisfaction with long-term condition management. To estimate these outcomes, the study used the same strategy described above for estimating the ARRS roles in the practice. The set of covariates were also the same as described above to estimate factors associated with the presence of FTE ARRS roles in practices. In both sets of analyses, the standard errors were robust and clustered at the practice level.

Stata (version 17.1) was used for all analyses.

## Results

**Figure 1.**
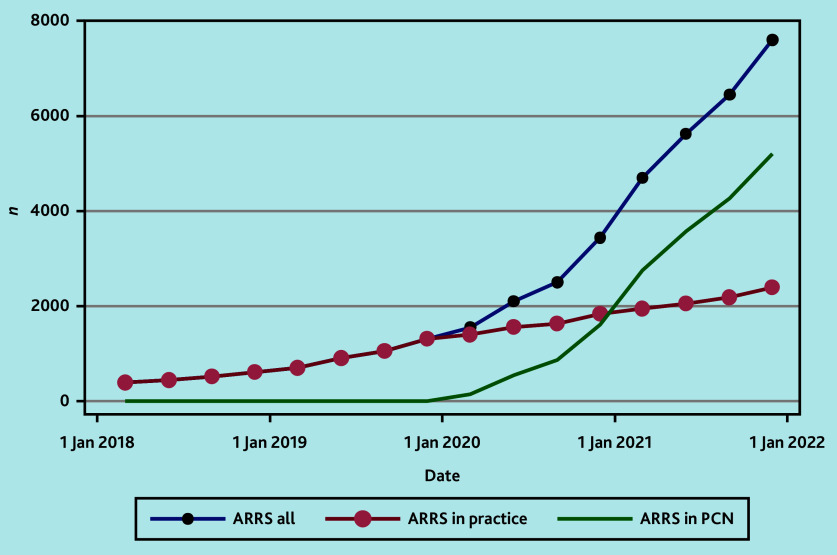
Trend across time of ARRS roles in general practices and PCNs. ARRS = Additional Roles Reimbursement Scheme. PCN = primary care network.

Figure 1 shows the trend of ARRS roles within general practices and PCNs across time. It was observed that prevalence of these roles increased after the NHS plan was introduced in 2019,^12^ with a greater expansion in PCN-employed ARRS roles rather than practice-employed ARRS roles since 2020.

Table 1 shows descriptive statistics for the key variables considered for both sets of analyses. On average, each general practice exclusively employed 0.122 FTE ARRS workers whereas an average of 0.327 FTE ARRS workers were employed either exclusively by the practice or through its PCN.

**Table 1.** Descriptive statistics for main study variables over the 2018–2022 study period^a^

**Variable**	**Mean**	**SD**
**FTE ARRS in practice**	0.122	0.327
**FTE ARSS practice and PCN**	0.327	0.759
**Number of patients weighted average**	8837.672	0.645
**FTE GP per 1000 patients**	0.565	0.284
**Rate of female GPs**	0.515	0.248
**FTE nurses per 1000 patients**	0.265	0.221
**Rate of overseas trained GPs**	0.342	0.319
**Rate of total prescription items per patient per year**	52.826	25.336
**Rate of prescriptions for mental health problems per patient per year**	1.475	0.536
**Rate of antibiotic prescriptions per patient per year**	0.659	0.256
**Rate of analgesic prescriptions per patient per year**	2.376	1.185
**Rate of statin prescriptions per patient per year**	0.110	0.046

a *Total observations,* n *= 106 453. ARRS = Additional Roles Reimbursement Scheme. FTE = full-time equivalent. PCN = primary care network. SD = standard deviation.*

**Figure 2.**
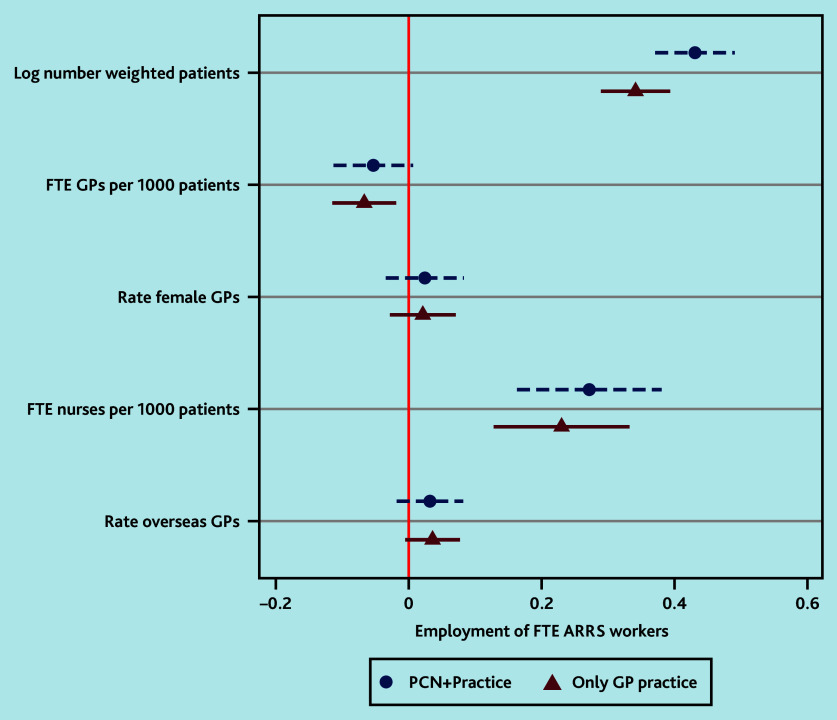
Coefficients plot for factors influencing employment of FTE ARRS workers in general practices in England. The regression models control for disease prevalence, CCG, year, and quarter fixed effects, with standard errors clustered at the practice level. ARRS = Additional Roles Reimbursement Scheme. CCG = clinical commissioning group. FTE = full-time equivalent. PCN = primary care network.

Figure 2 presents the coefficients and standard errors from the first regression analysis on the total FTE count for ARRS workers, including those employed either exclusively by the practice or through its PCN and practice. It was observed that practices with an increased weighted number of patients, that is, larger practices, and more FTE nurses and overseas trained GPs, tended to have more FTE ARRS workers (0.35, *P*<0.001, and 0.234 *P*<0.001, respectively, for staff employed through practices or through practices plus PCNs).

A 1 percentage point increase in the weighted list size of a practice increased the FTE count of ARRS workers by 0.35 points. ARRS workers appeared less frequently in practices with more FTE GPs per patient (−0.075, 95% confidence interval = −0.126 to −0.025, *P*<0.05) for the analysis of only practice and PCN plus practice, implying that they tended to be deployed in areas with some GP shortages (Figure 2). Rurality or socioeconomic deprivation were not controlled for in this analysis as this information was already incorporated into the patient list size weighting measure.

Table 2 reports the coefficients for the OLS regression analysis examining the association between ARRS roles and prescribing rates. Values are only presented for the FTE counts of ARRS workers employed through both general practices and PCNs. The study found that general practices with more ARRS-reimbursed workers had lower total prescription rates overall (−5.56, *P*<0.001). A 1 percentage point FTE increase was associated with an average 5 percentage point decrease in the rate of prescriptions. In particular, the employment of ARRS workers was associated with slightly lower rates of prescribing of mental health medications (−0.18, *P*<0.001).

**Table 2.** Regression analysis of the effect of ARRS workers on prescription rates at general practice level^a^

**Variable**	**Total prescriptions, β (SE)**	**Antibiotics, β (SE)**	**Statins, β (SE)**	**Mental health, β (SE)**	**Analgesics, β (SE)**
**FTE ARRS employed through general practices and PCNs**	−5.56^b^ (1.53)	−0.03 (0.03)	−0.01 (0.00)	−0.18^b^ (0.05)	−0.13 (0.07)
**Log patients weight average**	−48.82 (5.88)	−1.67^b^ (0.22)	−0.07^b^ (0.01)	−3.39^b^ (0.24)	−5.60^b^ (0.27)
**FTE GPs per 1000 patients**	1.05 (10.25)	0.63 (0.48)	0.03^c^ (0.01)	0.16 (0.22)	−0.84 (0.53)
**Rate of female GPs**	−7.94 (7.71)	−0.01 (0.07)	−0.04^d^ (0.01)	−0.23 (0.17)	−0.57 (0.36)
**FTE nurses per 1000 patients**	1.11 (0.69)	0.01 (0.02)	0.000 (0.00)	0.01 (0.01)	0.01 (0.01)
**Rate of overseas trained GPs**	4.17 (6.51)	−0.12 (0.08)	0.06^b^ (0.01)	−0.43^d^ (0.16)	−1.07^d^ (0.38)

a *The regression models control for disease prevalence, CCG, year, and quarter fixed effects, with standard errors clustered at the practice level.*

b P*<0.001.*

c P*<0.05.*

d P*<0.01.*

*ARRS = Additional Roles Reimbursement Scheme. CCG = clinical commissioning group. FTE = full-time equivalent. PCN = primary care network. SE = standard error.*

Finally, Table 3 shows the association between FTE counts of ARRS workers employed through general practices and PCNs and overall patient satisfaction, as well as satisfaction with long-term condition management. It was observed that general practices with more FTE ARRS-reimbursed workers tended to have higher patient satisfaction scores. Specifically, increased ARRS staffing by a 1 percentage point was associated with improved overall satisfaction by >3 points (3.587, *P*<0.001). There was also a positive relationship between the number of FTE ARRS-reimbursed workers and patient satisfaction with long-term condition management (8.242, *P*<0.001).

**Table 3.** Regression analysis of the effect of ARRS workers on patient satisfaction at practice level^a^

**Variable**	**Overall satisfaction, β (SE)**	**Satisfaction with management of chronic disease, β (SE)**
**FTE ARRS in practice and PCN**	3.587^b^ (−0.64)	8.242^b^ (−1.00)
**Log patients weighted average**	41.731^b^ (−2.73)	73.648^b^ (−4.52)
**FTE GPs per 1000 patients**	−11.641^b^ (−2.32)	5.235^c^ (−1.72)
**Rate female GPs**	1.569 (−2.00)	1.561 (−1.43)
**FTE nurses per 1000 patients**	−0.146^b^ (−0.04)	0.045 (−0.04)
**Rate of overseas trained GPs**	−0.854 (−1.70)	−2.685 (−1.62)
**FTE senior partner GPs per 1000**	−12.133 (−7.65)	11.968 (−7.57)

a *The regression models control for disease prevalence, CCG, year, and quarter fixed effects, with standard errors clustered at the practice level.*

b P*<0.001.*

c P*<0.01.*

*ARRS = Additional Roles Reimbursement Scheme. CCG = clinical commissioning group. FTE = full-time equivalent. PCN = primary care network.*

## Discussion

### Summary

This study analysed the presence of ARRS roles in primary care as well as the association between ARRS roles and prescribing patterns and patient satisfaction in English general practices. It was found that ARRS roles are employed more frequently in larger practices and practices with a shortage of GPs after controlling for the list size and indicators of patient needs. Additionally, general practices with more ARRS staff had lower prescribing rates, especially for mental health medications. ARRS roles were also linked to higher patient satisfaction overall and satisfaction with long-term condition management. These findings suggest that expanding the ARRS workforce as part of PCNs may help reduce elements of prescribing and improve patient experience, although further investigation is needed as these associations are not necessarily causal.

The lower prescribing rate could be attributed to the strong emphasis on adherence to guidelines in the training of advanced practitioners, and to the availability of a wider range of forms of help, which may reduce the need for prescribed medication. This is particularly consistent with the employment of a high number of clinical pharmacists. By providing more time with a broader care team, ARRS staff may improve satisfaction, especially for patients with ongoing health conditions requiring regular monitoring and coordination. However, further research should explore whether specific ARRS roles, such as health and wellbeing coaches, drive improvements in satisfaction with long-term condition management.

Overall, the current findings align with the goals of the ARRS programme to enhance capacity, quality, and access in primary care. Identifying barriers to implementation can inform policy and practice to maximise the benefits of this substantial workforce investment. The findings should provide needed insight into how ARRS is implemented and its potential to improve primary care delivery.

### Strengths and limitations

A strength of this study is the use of comprehensive national-level datasets to evaluate ARRS staffing levels, prescribing volumes, and patient satisfaction metrics longitudinally across thousands of general practices. However, there are limitations to note. The analysis relied on practice-level data, so the results are unlikely to represent individual-provider prescribing behaviours. It was not possible to account for key patient demographic and clinical factors, such as patient income, education, or multimorbidity that could influence prescribing patterns. There may also be unobserved confounding factors driving the observed associations, for example, practices that employ ARRS staff may be better organised in other ways that affect prescribing and patient satisfaction. The study also did not differentiate between the various types of ARRS staff across the practices. Although the authors observed several roles operating within the general practices, it was not possible to analyse the time spent or specific duties carried out by each individual role. Furthermore, the authors did not have information on how ARRS workers distributed their time across different general practices within PCNs. In the current analysis, the assumption was made that ARRS workers were evenly distributed across all practices within a PCN, allocating their working hours equally among the constituent practices. However, it is possible that the distribution of ARRS workers’ time may vary across practices within a PCN, depending on factors such as practice size, patient needs, or other organisational factors. Given the limitations of the cross-sectional design, the findings should be interpreted with caution. Further research is needed to explore the hypotheses generated by this study and to investigate potential causal relationships.

### Comparison with existing literature

Previous published evidence has demonstrated that higher nurse staffing levels in primary care settings correlated with improved processes and outcomes for chronic disease management. Increased nursing consultations have shown an association with better diabetes control in general practice. Additionally, certain primary care nursing workforce traits link to superior high blood pressure regulation.^13^^–^^15^

A recent study by Gibson and colleagues found that an increase in GPs was positively associated with both GP job satisfaction and patient satisfaction, while additional non-GP staff had the opposite effect on these outcomes.^16^ Evaluating the impact of ARRS roles on patient and health system outcomes has been limited to date.^17^^–^^21^ The current study provides some of the first empirical evidence, to the best of the authors’ knowledge, regarding how expanding the primary care skill-mix through these reimbursement-funded new roles may benefit patients and practices. The current results that show ARRS roles could decrease prescription rates are in line with the study by Riordan *et al* that found that pharmacist prescribing can decrease medication use.^22^ Early evaluations of social prescribing link workers suggest they may also reduce prescribing, particularly for mental health problems.^23^ The current findings showing a positive association between ARRS staffing levels and patient satisfaction align with recent work by Penfold *et al*.^24^ They found that higher FTE ARRS staffing levels were associated with an increase in the proportion of patients satisfied with their care. However, their analysis was limited to satisfaction with staff employed solely through PCNs and did not take into account staff employed directly by general practices.

The current study contributes novel national-level evidence across the range of ARRS roles on associations with prescribing rates and patient satisfaction.

### Implications for research and practice

General practices currently confront a number of decisions in considering the employment of various health professionals to, for instance, optimise skill-mix advantages for their population and adapt workloads to support GP recruitment and retention. For general practices, the current results suggest that investing in ARRS roles, especially those supporting mental health and long-term conditions, may help reduce prescribing and improve patient satisfaction. Policymakers should continue monitoring the rollout of the ARRS and strengthening training and collaboration for new primary care roles.

The integration of advanced clinical roles has the potential to ease a number of pressures faced by general practices. Their involvement could alleviate workforce shortages resulting from recruitment and retention challenges among GPs. However, critics argue there are also substantial downsides and open questions regarding the ARRS policy.^25^^–^^28^ Others argue the modest benefits found in studies like that by Penfold *et al* (2023) and Penfold *et al* (2024) may not justify the high costs of these new staff, plus ongoing investments in training and GP supervision time.^24^^,^^29^^,^^30^

Although it is unsurprising that adding more staff is likely to enhance patient experience owing to improved access and greater consultation time, this does not in itself guarantee the quality of care provided during consultations. It is also important to consider patient safety when evaluating the value of non-GP professionals in primary care, although difficult to measure. Ensuring proper training, guideline adherence, and outcome monitoring is essential to mitigate risks and provide high-quality care. Most ARRS staff have not undergone the same level of training, supervision, and formal assessment as GPs during their medical education, and some of these roles are not currently regulated. The absence of a standardised, primary care-specific training programme for some of the ARRS roles could be a key source of concerns regarding the safety implications of integrating these new workforce members into general practice settings. A recent study by Walsh *et al* found that first-contact physiotherapists offered safe and clinically effective management for patients with musculoskeletal disorder in general practice settings, but assessment of quality and safety should continue to be a priority for future research on expanding non-GP roles.^18^ More research is also needed to provide definitive return-on-investment analysis on the long-term costs, risks, and benefits of integrating ARRS workers compared with simply expanding doctor staffing capacity.

In conclusion, this study provides preliminary evidence that expanding primary care skill-mix through ARRS workforce investments may contribute to lower prescribing rates and improved patient satisfaction. However, substantial unknowns persist regarding their long-term return-on-investment, the ideal composition of roles, their impact on continuity and coordination, which patients are most appropriate for them to see, and potential risks from rapid integration of these new roles in primary care. These areas should be priorities for future research.

## Data

The analysis presented in this article utilises publicly accessible data.
